# The effect of pulsatile lavage on antibiotic elution from bone cements in two-stage revision for periprosthetic joint infection

**DOI:** 10.1186/s13018-025-06454-z

**Published:** 2025-11-19

**Authors:** Kevin Knappe, Mareike Schonhoff, Therese Bormann, Timo A. Nees, Andre Lunz, Sebastian Jaeger

**Affiliations:** 1 Department of Orthopaedics, Heidelberg University Hospital, Schlierbacher Landstrasse 200a, 69118 Heidelberg, Germany; 2 Research Center of Biomechanics and Implant Technology, Heidelberg University Hospital, Schlierbacher Landstrasse 200a, 69118 Heidelberg, Germany

**Keywords:** Periprosthetic joint infections PJI, Pulsatile lavage, Two-stage revision, Spacer, Antibiotic-loaded bone cement, Gentamicin, Vancomycin

## Abstract

**Purpose:**

Periprosthetic joint infection (PJI) is a major complication after total joint replacement, often requiring a two-stage revision with the use of antibiotic-loaded bone cement (ALBC) spacers for local antibiotic therapy. While factors such as cement composition and antibiotic type influence drug release, the impact of pulsatile lavage on antibiotic elution remains unclear. This study investigates the effect of pulsatile saline lavage on the release kinetics of gentamicin and vancomycin from different ALBCs over six weeks.

**Methods:**

Three commercially available PMMA bone cements A, B and C were prepared with identical antibiotic concentrations (0.5 g gentamicin, 2 g vancomycin). Rectangular cement blocks were manufactured according to ISO standards. Group A1/B1/C1 (non-lavage) was placed into a buffer solution without further processing. Group A2/B2/C2 (lavage) was treated with a defined amount of saline solution using high-pressure pulsatile saline lavage before being placed into the buffer solution. Antibiotic release was analyzed at ten predefined time points (T) over six weeks (5 h, 1 d, 2 d, 4 d, 1/2/3/4/5/6 weeks). All samples were stored in an incubator at 36 °C.

**Results:**

Statistically significant differences in the release kinetics of both gentamicin and vancomycin were observed for bone cement A and B after processing with saline lavage. Gentamicin release differed significantly between A1/A2 at T2 and T7–T10, and between B1/B2 at T1, T7, and T10; no significant differences were found between C1/C2. Cumulatively, only B1 vs. B2 showed a significant difference in gentamicin release (*p* = 0.033). Vancomycin release differed significantly between A1/A2 at T1 and T5–T9, and between B1/B2 at T6; again, no significant differences were seen in C1/C2. Cumulative vancomycin release was significantly reduced in B2 compared to B1 (*p* < 0.001). In four of six cements, vancomycin release declined by over 85% from T1 (5 h) to T3 (48 h).

**Conclusion:**

Pulsatile high-pressure lavage is an essential part of revision surgery due to its mechanical cleansing effects. However, this study indicates that its impact on antibiotic release from loaded bone cement is minimal. While variations exist depending on cement type, lavage does not seem to substantially alter the elution profile or expected antimicrobial efficacy.

## Introduction

**Fig. 1:**
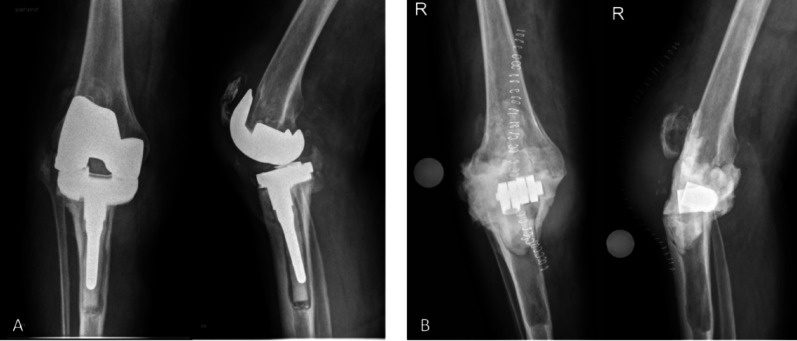
**A** Preoperative radiographs demonstrating septic loosening of a total knee arthroplasty. **B** Postoperative radiograph after prosthesis removal and implantation of an arthrodesis bone cement spacer

Periprosthetic joint infection (PJI) is a feared complication after total joint replacement. In the annual report of the American Joint Replacement Registry from 2024, it is the most common cause of revision surgery [1]. The global gold standard in the treatment of chronic PJI is a two-stage revision [2–5]. The first stage involves removal of the prosthesis and, if necessary, bone cement, followed by radical debridement of bone and soft tissues. Local antiseptics and copious isotonic irrigation are applied using various pulsatile lavage systems, which differ in flow rate, pulse frequency, and flushing pressure. [6, 7]. To provide local antibiotic therapy it is widely recommended to use an antibiotic-loaded bone cement (ALBC) spacer for the interim period (Fig. 1). After about 6–12 weeks, another radical debridement is carried out in the second step and a new prosthesis is implanted [8, 9]. It is known that intraoperative addition of antibiotics to the bone cement results in higher local antibiotic concentrations and thus presumably a more effective treatment of the infection [10–13]. In addition, the bone cement spacer conditions the periarticular soft tissues for later reimplantation. When designed as an articulating spacer, it also provides good functional outcomes and high patient satisfaction rates [14–16]. Ideally, sustained high local antibiotic release is achieved until reimplantation, providing effective intra-articular concentrations while minimizing systemic side effects. Different factors, such as the bone cement, the amount and type of antibiotics added and the size of the surface area critically influence the antibiotic release [12, 17–19]. High-pressure pulsatile saline lavage has been shown to be an effective technique for reducing bacterial contamination on various surfaces, leading to a significant decrease in bacterial load across different types of wounds [20–22]. It has also been demonstrated that pulsatile jet lavage is more effective than a bulb syringe alone in reducing bacterial load and removing necrotic tissue and foreign particles from wounds [23–25]. Therefore, even after thorough debridement, lavage, and antibiotic spacer implantation, performing high-pressure pulsatile lavage to further clean the surgical site represents an effective and technically straightforward approach. However, it remains unclear whether the use of high-pressure pulsatile lavage has an influence on the antibiotic release of the antibiotic loaded bone cement and thus on the local antibiotic concentration.

Therefore, this study aimed to examine different antibiotic-loaded bone cements after processing them with pulsatile lavage for their release of gentamicin and vancomycin over a period of 6 weeks.

## Methods

In this study three different bone cements were used. Each bone cement group was examined without and after pulsatile lavage application. This resulted in a total of six study groups (Table 1).

**Table 1:** Comparison of the three different bone cements and their concentration of antibiotics in 40 g of bone cement

Group	Bone cement	Premixed gentamicin	Premixed vancomycin	Addition of gentamicin	Addition of vancomycin	Pulsatile lavage
A1	Palacos R + G	0.5 g	–	-	2 g	-
A2	Palacos R + G	0.5 g	–	–	2 g	Yes
B1	Copal G + V	0.5 g	2 g	–	–	–
B2	Copal G + V	0.5 g	2 g	–	–	Yes
C1	Copal spacem	–	–	0.5 g	2 g	–
C2	Copal spacem	–	–	0.5 g	2 g	Yes

### Bone cement A

Palacos R + G (Heraeus Medical, Wehrheim, Germany) already contains 0.5 g of gentamicin. In addition, 2 g of vancomycin were added per 40 g of bone cement (Polymer powder ingredients in the size 40: 41.3 g powder: Poly(methyl acrylate, methyl methacrylate) PMMA 82%, zirconium dioxide 15%, hydrous benzoyl peroxide 1%, gentamicin sulfate 2%. 20 ml monomer liquid ingredients: methyl methacrylate 98%, N,N-dimethyl-p-toluidine 2%. Other constituents: In the cement powder: chlorophyll VIII (colorant E141)and in the monomer liquid: chlorophyll VIII (colorant E141) in an oily solution, hydroquinone)) [26].

### Bone cement B

Copal G + V (Heraeus Medical, Wehrheim, Germany) already contains 0.5 g gentamicin and 2 g vancomycin (Ingredients/Composition in the size 40: 43 g powder: PMMA copolymer 78%, zirconium dioxide 14%, benzoyl peroxide 1%, gentamicin sulfate 2%, vancomycin hydrochloride 5%. 20 ml Liquid: methyl methacrylate 98%, N,N-dimethyl-p-toluidine 2%. Other constituents: chlorophyll-copper-complex (E141)) [27]. It was used as distributed by the manufacturer.

### Bone cement C

Copal spacem (Heraeus Medical, Wehrheim, Germany) is supplied without added antibiotics (Polymer powder ingredients: Poly(methylacrylate, methyl methacrylate, zirconium dioxide, benzoyl peroxide and colorant E141. Monomer liquid ingredients: Methyl methacrylate, N,N-dimethyl-p-toluidine, hydroquinone and colorant E141.) [28]. Here, both 0.5 g of gentamicin and 2 g of vancomycin were added per 40 g of bone cement.

**Fig. 2:**
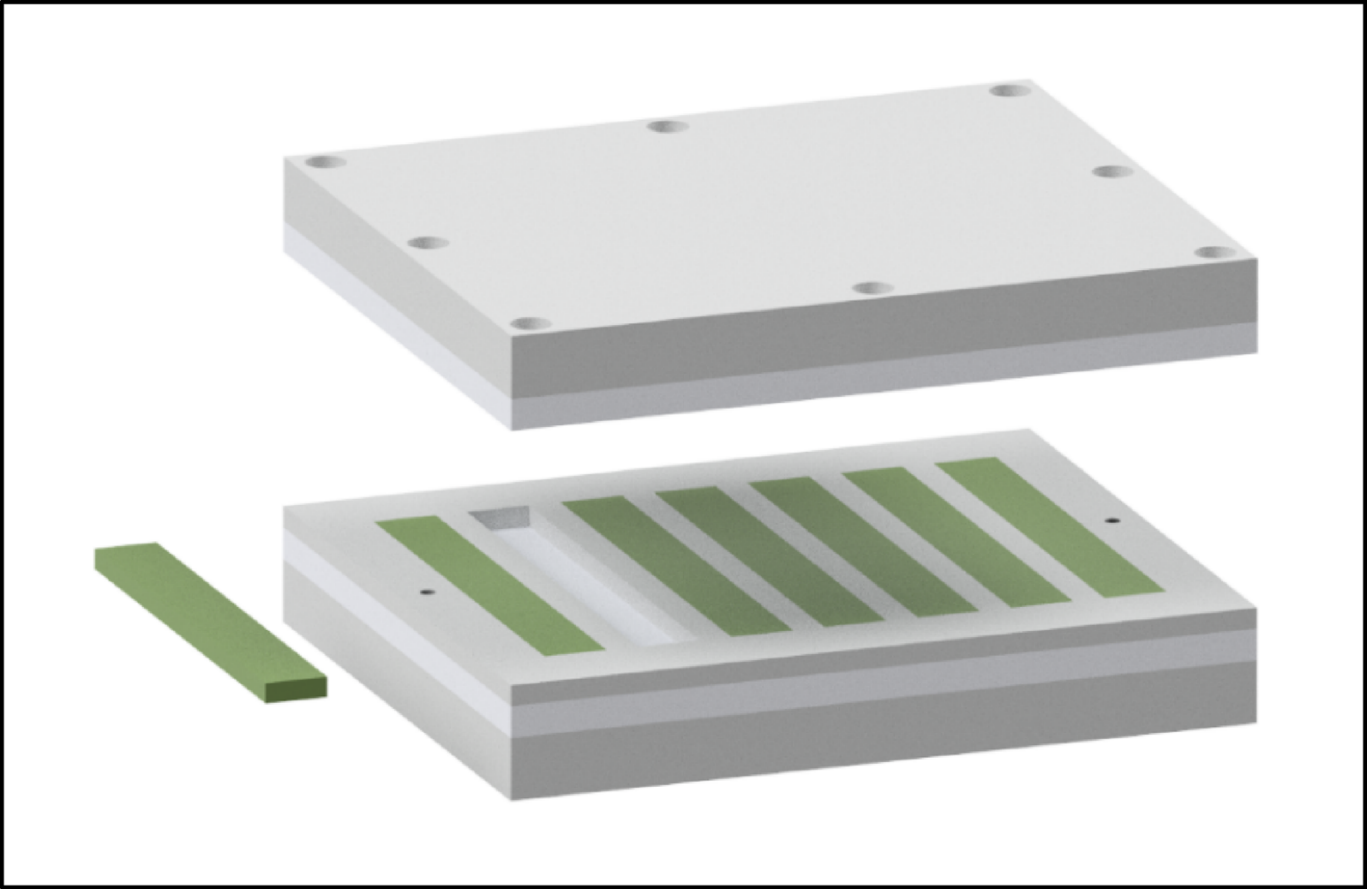
Mold designed to produce seven standardized rectangular blocks, measuring 3.3 × 10 × 75 mm, in accordance with ISO 5833:2002. After insertion of the bone cement, the components are assembled, the device is securely sealed, and polymerization is awaited

Thus, all 3 cements contained the same amount of gentamicin and vancomycin. All cement mixing was carried out at room temperature (23 ± 1 °C) and a minimum humidity of 40%, without the use of a vacuum. Precisely 60 s after the start of bone cement polymerization, the cement was applied into the mold using a cement gun (Fig. 2). The mold was designed to produce seven standardized rectangular blocks, each measuring 3.3 × 10 × 75 mm, in accordance with ISO 5833:2002, for each spacer group. The samples were additionally examined radiologically for obstructive air inclusions. This method has already been applied in numerous publications [12, 29, 30].

The samples in group 1 (A1 with bone cement A/B1 with bone cement B/C1 with bone cement C) were then placed individually in 40 ml of phosphate-buffered saline (PBS) with a pH of 7.4. Incubation took place at 37 °C.

The samples in group 2 (A2 with bone cement A/B2 with bone cement B/C2 with bone cement C) were processed from both sides immediately after the cement was cured using a commercially available, single-use pulsatile lavage device (InterPulse, Stryker, Kalamazoo, Michigan, USA) to ensure reproducibility. The device operated at 0.53 ± 0.02 N/mm^2^ with a flow rate of 0.65 ± 0.001 L/min, a pulse frequency of 19.86 ± 1.07 Hz, and a standardized nozzle distance of 15 mm using the splash shield. The spray attachment featured four straight, non-angled nozzles, each with a 1 mm^2^ cross-sectional area [6]. A total of 200 mL irrigation fluid was applied per specimen, divided equally between the upper and lower surfaces (10 × 75 mm each).

Antibiotic concentrations were measured using a chemiluminescent immunoassay (Advia Centaur XPT, Siemens Healthineers, Germany) at 10 predefined time points (T1: five hours, T2: one day, T3: two days, T4: four days, T5: one week, T6: two weeks, T7: three weeks, T8: four weeks, T9: five weeks and T10: six weeks). The entire medium was replaced at each measurement time point for each probe. With five samples per group across six groups and ten time points, a total of 300 samples were analyzed for gentamicin and vancomycin concentrations. The measurement limits for gentamicin were 0.5 and 24.0 mg/l. For vancomycin, only the lower detection limit of 3.0 mg/l was reached. Accordingly, the limit values mentioned were adopted as measurement values. An ethics approval was not necessary for this in vitro study.

### Statistical analysis

Descriptive data is shown as the arithmetic mean and standard deviation. Normal distribution of the data could be shown using the Shapiro–Wilk test. Since there was no sphericity in a mixed ANOVA, a correction following Greenhouse- Geissler was applied.

Homogeneity of error variances between groups was met for all variables according to the Levene test (*p* > 0.05), so a univariate ANOVA according to Welch was run.

Last, a Bonferroni-adjusted post-hoc analysis revealed the significant differences between groups 1 and 2 (A1 vs. A2; B1 vs. B2; C1 vs. C2) at the ten different time points.

The level of significance was set at *p* < 0.05 for all statistical tests. The statistical analyses were performed using the software SPSS (version 25.0; IBM Inc., Armonk, New York, NY, USA).

## Results

### Comparison of gentamicin release in non-lavage and lavage group (Fig. 3)

**Fig. 3:**
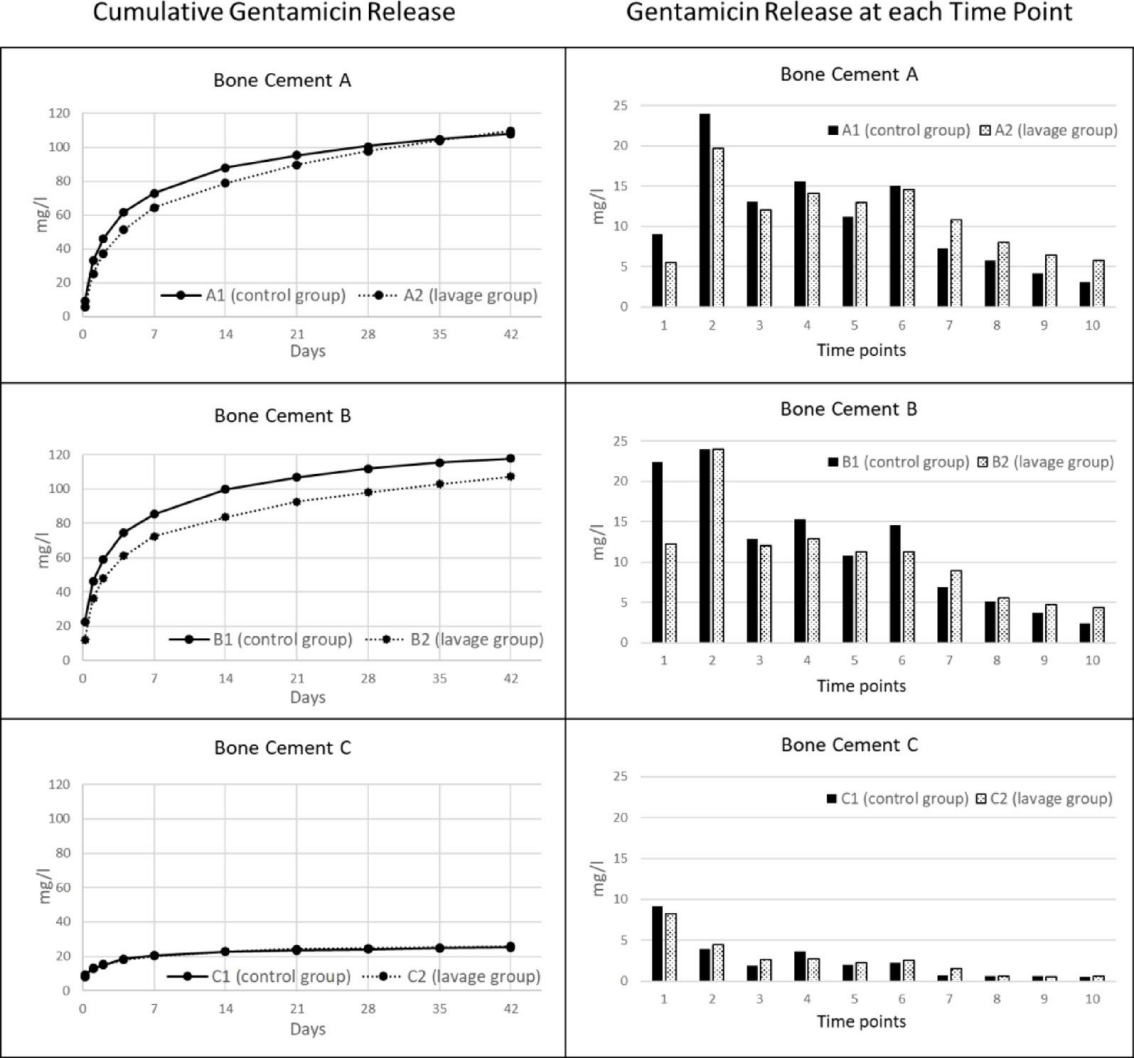
Left: Mean cumulated gentamicin release over time of bone cement A, B and C in mg/l. Right: Gentamicin release in mg/l at time points 1–10 (1: five hours, 2: one day, 3: two days, 4: four days, 5: one week, 6: two weeks, 7: three weeks, 8: four weeks, 9: five weeks, 10: six weeks)

Significant differences could be shown between the groups A1/B1/C1 (non-lavage) and A2/B2/C2 (lavage) over all time points T1 to T10 (F(9.45, 45.34) = 24.59, *p* < 0.001).

Group A1 and A2 showed statistically significant differences in their gentamicin release at the time points T2 (one day) and T7-10 (three, four, five and six weeks).

Group B1 and B2 showed statistically significant differences in their gentamicin release at the time points T1, T7 and T10.

Group C1 and C2 showed no significant differences in their gentamicin release over all time points.

For the cumulative gentamicin release over the entire six weeks, there was a significant difference between groups B1 vs. B2 (t(8) = 2.582; *p* = 0.033), no significance between groups A1 vs. A2 (t(8) = 0.701; *p* = 0.503) and C1 vs. C2 (t(8) = − 0.652; *p* = 0.532) (Table 4).

### Comparison of vancomycin release in non-lavage and lavage group (Fig. 4)

**Fig. 4:**
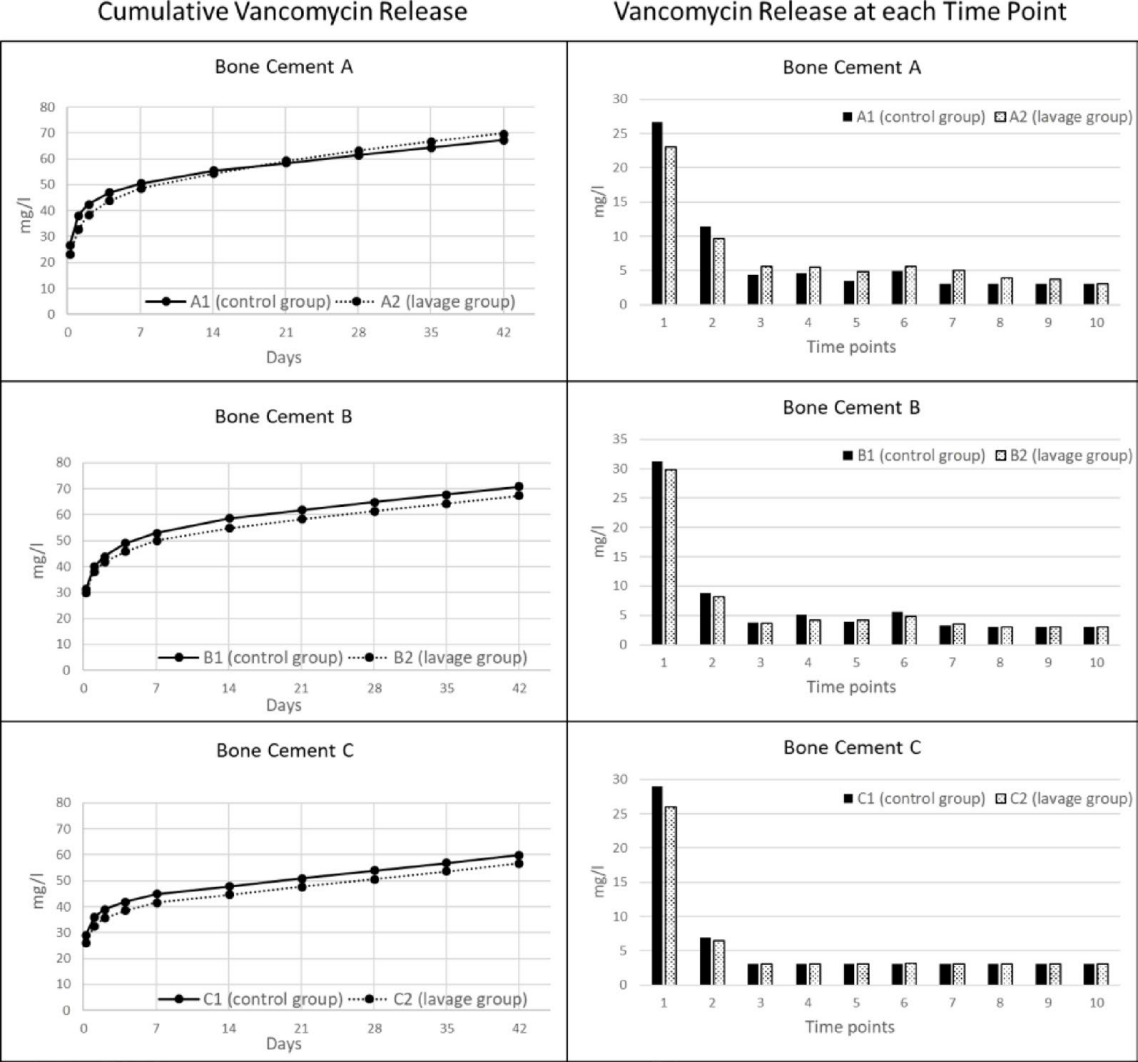
Left: Mean cumulated vancomycin release over time of bone cement A, B and C in mg/l. Right: Vancomycin release in mg/l at time points 1–10 (1: five hours, 2: one day, 3: two days, 4: four days, 5: one week, 6: two weeks, 7: three weeks, 8: four weeks, 9: five weeks, 10: six weeks)

Significant differences between group 1 (A1, B1, C1: no lavage) and group 2 (A2, B2, C2: lavage) could be shown F(11.04, 52.99) = 16.76, *p* < 0.001.

Group A1 and A2 showed statistically significant differences in their vancomycin release at the time points T1 (5 h) and T5—9 (one, two, three, four, five weeks). Group B1 and B2 showed statistically significant differences in their vancomycin release at the time point T6 (two weeks).

Group C1 and C2 showed no significant differences in their vancomycin release over all time points. The measurement limits for gentamicin were reached in group A1, B1 and B2 at time point T2 (one day), in group C1 at T10 (six weeks) and in group C2 at T9 (five weeks) (Table 2).

**Table 2:** Mean gentamicin release with standard deviation (T1- five hours, T2- one day, T3- two days, T4- four days, T5- one week, T6- two weeks, T7- three weeks, T8- four weeks, T9- five weeks and T10- six weeks). A1/B1/C1 no lavage, A2/B2/C3 with lavage (A1/2- Palacos R + G, B1/2- Copal G + V, C1/2- Copal spacem)

Gentamicin (mg/l)										
Group	T1	T2	T3	T4	T5	T6	T7	T8	T9	T10
A1	9.1 ± 1.3	24.0 ± 0.0	13.0 ± 1.0	15.6 ± 1.4	11.2 ± 0.7	15.0 ± 0.9	7.3 ± 1.0	5.8 ± 0.7	4.2 ± 0.7	3.1 ± 0.5
A2	5.5 ± 1.5	19.7 ± 3.3	12.1 ± 1.7	14.1 ± 2.1	12.9 ± 1.9	14.5 ± 1.9	10.8 ± 0.5	8.1 ± 0.7	6.5 ± 0.8	5.8 ± 0.9
*p*-value	1.0	0.003	1.0	1.0	0.249	1.0	< 0.001	0.001	< 0.001	< 0.001
B1	22.4 ± 2.9	24.0 ± 0.0	12.8 ± 2.1	15.3 ± 2.0	10.8 ± 1.2	14.5 ± 3.4	6.9 ± 1.5	5.1 ± 1.1	3.7 ± 1.0	2.4 ± 0.3
B2	12.2 ± 5.0	24.0 ± 0.0	12.0 ± 1.5	12.9 ± 1.8	11.3 ± 1.0	11.2 ± 0.9	8.9 ± 1.3	5.6 ± 0.9	4.7 ± 0.6	4.3 ± 0.5
*p*-value	0.0	1.0	1.0	0.420	1.0	0.076	0.042	1.0	0.264	< 0.001
C1	9.2 ± 0.6	3.9 ± 0.3	1.9 ± 0.4	3.6 ± 1.5	2.0 ± 0.1	2.2 ± 0.3	0.7 ± 0.1	0.6 ± 0.2	0.6 ± 0.3	0.5 ± 0.0
C2	8.2 ± 0.7	4.4 ± 2.0	2.7 ± 0.6	2.7 ± 0.4	2.2 ± 0.1	2.5 ± 0.8	1.5 ± 0.6	0.6 ± 0.1	0.5 ± 0.0	0.6 ± 0.2
*p*-value	1.0	1.0	1.0	1.0	1.0	1.0	1.0	1.0	1.0	1.0

The amount of vancomycin released decreased by more than 85% in four of the six cements examined from time T1 (5 h) to time T3 (two days). The decrease in release in the different groups was as follows: A1- 83.9%, A2- 76%; B1- 87.5%, B2- 88%; C1- 89.7%, C2- 88.5%.

At 27 of the 60 measurement times, the vancomycin concentration was below the detection limit of 3.0 mg/l (Table 3).

**Table 3:** Mean vancomycin release with standard deviation (T1- five hours, T2- one day, T3- two days, T4- four days, T5- one week, T6- two weeks, T7- three weeks, T8- four weeks, T9- five weeks and T10- six weeks). A1/B1/C1 no lavage, A2/B2/C3 with lavage (A1/2- Palacos R + G, B1/2- Copal G + V, C1/2- Copal spacem)

	Vancomycin (mg/l)									
Group	T1	T2	T3	T4	T5	T6	T7	T8	T9	T10
A1	26.7 ± 0.7	11.4 ± 0.9	4.3 ± 0.9	4.6 ± 0.7	3.5 ± 0.4	4.9 ± 0.3	3.0 ± 0.0	3.0 ± 0.0	3.0 ± 0.0	3.0 ± 0.0
A2	23.1 ± 2.4	9.7 ± 1.5	5.6 ± 2.2	5.4 ± 1.5	4.9 ± 1.1	5.6 ± 0.4	5.0 ± 0.4	3.9 ± 0.3	3.7 ± 0.3	3.0 ± 0.0
*p*-value	0.023	0.097	0.721	1.0	0.004	0.015	< 0.001	< 0.001	< 0.001	1.0
B1	31.2 ± 0.7	8.8 ± 0.6	3.9 ± 0.3	5.1 ± 0.2	4.0 ± 0.3	5.6 ± 0.4	3.3 ± 0.2	3.0 ± 0.0	3.0 ± 0.0	3.0 ± 0.0
B2	29.8 ± 0.9	8.2 ± 0.6	3.6 ± 0.2	4.2 ± 0.2	4.2 ± 0.2	4.8 ± 0.2	3.5 ± 0.3	3.0 ± 0.0	3.0 ± 0.0	3.0 ± 0.0
*p*-value	1.0	1.0	1.0	0.621	1.0	0.004	1.0	1.0	1.0	1.0
C1	29.0 ± 2.4	6.9 ± 0.8	3.0 ± 0.0	3.0 ± 0.0	3.0 ± 0.0	3.0 ± 0.0	3.0 ± 0.0	3.0 ± 0.0	3.0 ± 0.0	3.0 ± 0.0
C2	26.0 ± 1.5	6.5 ± 0.8	3.0 ± 0.0	3.0 ± 0.0	3.0 ± 0.0	3.0 ± 0.3	3.0 ± 0.0	3.0 ± 0.0	3.0 ± 0.0	3.0 ± 0.0
*p*-value	0.096	1.0	1.0	1.0	1.0	1.0	1.0	1.0	1.0	1.0

As with gentamicin, there was a significant difference in the cumulative vancomycin release over the entire six weeks in groups B1 vs. B2 (t(8) = 8.130, *p* < 0.001), but no significance between groups A1 vs. A2 (t(8) = − 0.703, *p* = 0.502) and C1 vs. C2 (t(8) = 1.902, *p* = 0.094) (Table 4).

**Table 4:** Cumulative antibiotic release after 6 weeks (bone cement A1- control group, A2- lavage group; bone cement B1- control group, B2- lavage group; bone cement C1- control group, C2- lavage group)

	A1	A2	B1	B2	C1	C2
Gentamicin (mg/l)	113.0 ± 5.5	109.9 ± 8.2	127.7 ± 12.7	112.1 ± 4.7	25.3 ± 1.3	25.9 ± 1.9
Vancomycin (mg/l)	67.4 ± 3.1	69.8 ± 7.0	70.8 ± 0.4	67.4 ± 0.9	59.9 ± 2.9	56.6 ± 2.5

## Discussion

Several studies demonstrate that cleaning cancellous bone and contaminated wounds with pulsatile lavage is more effective than conventional irrigation [22, 31–34]. Pulsed lavage could also be shown to reduce the biofilm of Staphylococcus aureus effectively, but bacterial regrowth to baseline levels was seen after 24 h [35]. It is therefore important to combine the cleaning of contaminated tissue with the antimicrobial effect of an antibiotic [36].

It is well known that bone cement can be loaded with antibiotics, which are then effectively released from the cement [37–39]. But overall, there is varying data on the actual efficacy of antibiotic-loaded bone cements with regard to the prevention of postoperative infections in primary arthroplasty [40–43]. Gentamicin, as an aminoglycoside is primarily effective against gram-negative bacteria. Combining it with vancomycin, which is only effective against gram-positive pathogens, leads to high synergies and therefore is a frequently used combination. High peak concentration of gentamicin in relation to the minimal inhibitory concentration [44] is a major factor for the treatment for the infecting organism [45]. In systemic therapy with gentamicin, a serum concentration of more than 1 mg/l is associated with higher rates of renal dysfunction and even a higher mortality [46–48]. There have also been reported cases of renal failure using antibiotic-impregnated bone cement [49]. The recommendation in this regard is therefore to keep serum concentrations of gentamicin below 0.5–1 mg/l [50]. There are also reported cases of renal failure that seem to be related with vancomycin loaded bone cement [51, 52]. This contrasts with a study in which high doses of vancomycin were applied intraoperatively to the knee. Intraarticular vancomycin levels were measured from wound drainage fluid, while systemic levels were determined from blood serum. Although the synovial fluid values averaged up to 550 mg/l vancomycin on the first day and almost 80 mg/l on the fifth day, the serum concentration undercut the detection limit of 2.0 mg/l on the first day [53]. This is consistent with other studies that detected high local and low systemic concentrations after local application of vancomycin [54, 55]. For the intra-articular application of gentamycin, it was also demonstrated early on that this leads to high intra-articular and low serum and urine concentrations [56]. The same applies to the local application of gentamicin in the bone [57]. Based on these and other studies, there are no negative systemic effects of local gentamicin and vancomycin administration [58].

Since the effect of the respective antibiotic and the resulting minimum inhibitory concentration depends on the respective bacterium, it is difficult to give a generally valid dose or concentration recommendation that should be achieved intraarticularly. The most common bacteria in periprosthetic infections of hip and knee prostheses are coagulase-negative staphylococci, particularly Staphylococcus epidermidis, and Staphylococcus aureus. Together, these two pathogens account for the majority of infections, with coagulase-negative staphylococci responsible for approximately 30–43% of cases and Staphylococcus aureus for approximately 12–24% of cases [59–62]. For vancomycin the MIC to treat S. aureus seems to be around 1.0 mg/l [63, 64]. Sorensen et al. tested for the bactericidal activity of gentamicin against S. aureus and saw a MIC of 0.125–0.25 mg/l [65]. To prevent the selection of resistant subpopulations of bacteria, aminoglycosides can require about tenfold higher drug concentrations than the MIC [66]. Taken this into account, a gentamicin concentration of 2.0 mg/l seems to be effective [45]. Both groups A1/A2 and B1/B2 maintained levels above this threshold throughout the six-week period. In contrast, group C1/2 dropped below this level starting at T7 (3 weeks).

There are multiple reviews that focus on application, dosing and monitoring algorithms for intravenous vancomycin [63, 67–69]. If a minimum inhibitory concentration of 3.0 mg/l is taken as the lowest value for the effective treatment of germs with vancomycin, since this was the lower detection limit, this value is undercut in both the non-lavage and lavage groups after a similar time. In groups C1 and C2, this is already the case at examination time T3 (2 days). With this cement, the clinical benefit of added vancomycin appears questionable. However, it remains unclear how the dose levels in the patient change over time. In general, Spacem cement (group C), to which both antibiotics were added, has by far the lowest antibiotic release across all groups and time points, whereby the amount of antibiotics released in the medium does not change significantly at any time due to the jet lavage. Nevertheless, it is not clear if the low doses of antibiotics released might be enough to treat bacterial infections effectively.

The elution of gentamicin and vancomycin at the individual time points decreases continuously in all lavage groups. However, looking at the release of gentamicin in group 1 over time and compare the control group (A1) with the lavage group (A2), the release of antibiotics dissolved in the cement appears to be reversed. The lavaged samples initially release less antibiotics and then show a higher release over time. It is possible that the antibiotic is dissolved from deeper layers. Bone cement can absorb approx. 2% of liquid [70], but PMMA is generally a non-cancellous, hydrophobic material. In recent findings, a strong relationship of the porosity of the bone cement, which is higher with higher antibiotic doses and higher doses of radiopacifiers, and the elution rate of gentamicin could be shown [71]. So, higher porosity might lead to higher absorption of liquid and therefore potentially can wash out more of the antibiotics, as seen in group B. However, it is unclear why the reverse effect of more antibiotic elution at later time points does not occur in groups B and C. Falling below the detection limit appears to be a possible explanation here.

In general, the effect of pulsatile lavage on the release of antibiotics from the bone cement over the total period of six weeks only appears to be evident in prefabricated group B cement. With Copal cement (group B), which already contains gentamicin and vancomycin, a significant reduction in the cumulative release of antibiotics was measured and thus a presumably poorer effect in terms of bacterial treatment. This could be related to the more even/homogeneous distribution of the antibiotics already added industrially. Overall, this cement shows the most antibiotic release for both gentamicin and vancomycin. Even though lavage significantly reduces the gentamicin concentration in cement B, comparable concentrations to group A1 and more than fourfold to group C1 are achieved (Table 4). Surprisingly, gentamicin that is added manually (group C) to the cement appears to dissolve much less than when it has already been added industrially (group A/B). A significant influencing factor here could be the poorer homogenization during manual mixing in the operating theatre.

Even though the initial antibiotic release appears to be reduced, this analysis provides a scientific basis for the general assumption that the use of pulsatile saline high-pressure lavage has only a minor effect on the concentration and thus on the efficacy of added antibiotics.

Limitation: This is an in-vitro study where no metabolism takes place. Also, there is no movement of fluids, tissue or as in case of an articulating spacer movement of the components. Furthermore, it remains unclear if the standardized bone cement blocks and the used 40 ml of phosphate-buffered saline (PBS) are in a correct ratio to each other, compared to an in-vivo situation. A further limitation is that only antibiotic concentrations were measured, not the actual antibacterial effect. Also, the concentrations needed to effectively treat a PJI are very hard to discuss, since there are no large standardized clinical trials on local intraarticular concentrations. But as the study was designed to evaluate, if pulsatile lavage makes a difference in the antibiotic elution from impregnated bone cement, this experimental setup shows a high level of standardization and reproducibility.

## Conclusion

Pulsatile high-pressure lavage remains an indispensable component of revision arthroplasty, particularly due to its proven efficacy in removing debris and biofilm from contaminated surgical sites. The results of this in-vitro study suggest that its influence on the release kinetics of antibiotics from impregnated bone cement is low. While minor variations in antibiotic elution were observed—particularly in prefabricated cements with industrially mixed agents—the overall impact of pulsatile lavage on antibiotic concentrations over time appears to be minimal.

## Data Availability

The dataset used and/or analyzed during the current study is available from the corresponding author upon reasonable request.
